# Assessing health centre systems for guiding improvement in diabetes care

**DOI:** 10.1186/1472-6963-5-56

**Published:** 2005-08-24

**Authors:** Damin Si, Ross Bailie, Christine Connors, Michelle Dowden, Allison Stewart, Gary Robinson, Joan Cunningham, Tarun Weeramanthri

**Affiliations:** 1 Menzies School of Health Research, Charles Darwin University, PO Box 41096, Darwin, NT, Australia; 2 Northern Territory Department of Health and Community Services, Darwin, NT, Australia; 3 School for Social and Policy Research, Charles Darwin University, Darwin, NT, Australia

## Abstract

**Background:**

Aboriginal people in Australia experience the highest prevalence of diabetes in the country, an excess of preventable complications and early death. There is increasing evidence demonstrating the importance of healthcare systems for improvement of chronic illness care. The aims of this study were to assess the status of systems for chronic illness care in Aboriginal community health centres, and to explore whether more developed systems were associated with better quality of diabetes care.

**Methods:**

This cross-sectional study was conducted in 12 Aboriginal community health centres in the Northern Territory of Australia. Assessment of Chronic Illness Care scale was adapted to measure system development in health centres, and administered by interview with health centre staff and managers. Based on a random sample of 295 clinical records from attending clients with diagnosed type 2 diabetes, processes of diabetes care were measured by rating of health service delivery against best-practice guidelines. Intermediate outcomes included the control of HbA1c, blood pressure, and total cholesterol.

**Results:**

Health centre systems were in the low to mid-range of development and had distinct areas of strength and weakness. Four of the six system components were independently associated with quality of diabetes care: an increase of 1 unit of score for organisational influence, community linkages, and clinical information systems, respectively, was associated with 4.3%, 3.8%, and 4.5% improvement in adherence to process standards; likewise, organisational influence, delivery system design and clinical information systems were related to control of HbA1c, blood pressure, and total cholesterol.

**Conclusion:**

The state of development of health centre systems is reflected in quality of care outcome measures for patients. The health centre systems assessment tool should be useful in assessing and guiding development of systems for improvement of diabetes care in similar settings in Australia and internationally.

## Background

Indigenous Australians experience the highest prevalence of diabetes in the country, an excess of preventable complications and early death [1]. Published studies have demonstrated that effective diabetes management in Indigenous primary care can improve the process and outcomes of care [2-4]. Reported approaches to improving quality of care include use of recall and reminder systems, audit and feedback of clinical performance, structured clinical care, and specialist involvement in primary care. This evidence on the effectiveness of approaches to care in the Indigenous Australian population is consistent with the approach reflected in the Chronic Care Model developed in the USA [5,6]. The Chronic Care Model is comprised of six major components that have been shown to be important internationally to chronic illness care: 1) health care organisation, 2) community linkages, 3) self-management support, 4) decision support, 5) delivery system design, and 6) clinical information systems. This model has been extensively implemented in community health centres and hospitals in the USA to assess system support for chronic care and to identify areas for further improvement [7].

In the context of Australia's Northern Territory (NT) health service providers' collaborative efforts to improve prevention and management of chronic disease in primary health care [8], the Audit and Best practice for Chronic Disease (ABCD) project was implemented on the expectation that community health centre staff and managers would benefit from an improved understanding of the status of health centre systems in order to appropriately plan for improvement. A central component of the ABCD project was therefore an assessment of health centre systems as outlined in the Chronic Care Model. This paper reports on the association of the systems assessment with the quality of diabetes care for ABCD participating centres at baseline.

## Methods

### Study design, setting and selection of participating health centres

**Figure 1:**
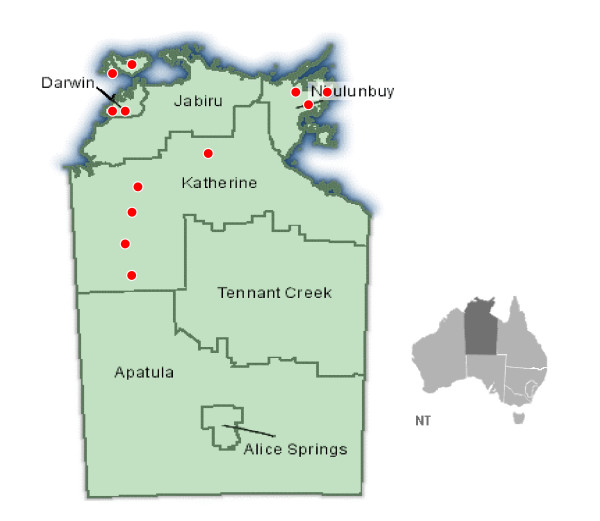
Distribution of 12 participating communities in the Top End of the Northern Territory, Australia.

This cross-sectional study was conducted in the Top End, an area covering one-third of NT. Of 60 Aboriginal communities with a population of 50 or more that are located in this area, 45 have a health centre within the community [9]. The selection of 12 health centres for participation in the study aimed to reflect the range in size, geographic location and governance arrangements existing in Top End communities (Figure 1). Community populations ranged from 180 to 1500. Five community health centres were managed by the NT Department of Health and Community Services, four by Aboriginal Health Boards, and three by Aboriginal Medical Services.

Community members who met all of the following criteria were included in the study: 1) a definite diagnosis of type 2 diabetes according to health centre records; 2) identified as Aboriginal; 3) aged 16 years or older; and 4) lived in the community for 6 months or more during the previous 12 months. A random sample of 30 records was drawn from four of the twelve community health centres where more than 30 eligible people were identified. In the other eight centres the records of all eligible people were included.

### Measurement and data collection

#### System mapping

The Assessment of Chronic Illness Care (ACIC, Version 3.5) scale [10], a practical tool based on the Chronic Care Model [11], was used to evaluate the status of health centre systems to support chronic illness care. A minor adaptation was made to facilitate its use in the local setting. The adapted scale consists of 34 items covering the six components of the Chronic Care Model and an additional domain which denotes the level of integration of the six components: health care organisation (3 items), community linkages (4 items), self-management (4 items), clinical decision support (3 items), clinical delivery system (9 items), clinical information system (6 items), and integration (5 items). Compared with the original ACIC scale, this adapted version included three additional items (cultural competence, pathology management, and pharmacy management) in the clinical delivery system domain, to reflect specific features of interest in NT centres.

A group of health centre staff (manager, doctor, nurse, and/or Aboriginal Health Worker when available) was guided by researchers on how to complete the scale. The answer to each item in the adapted scale requires recording of a score in the range of 0–11. The scale includes a set of prompts (see Additional file 1) to increase standardisation and reproducibility in scoring and staff are requested to provide a qualitative justification for their score in relation to these prompts (see example in Additional file 2). The scores are categorised as: 0–2 (limited support); 3–5 (basic support); 6–8 (good support); and 9–11 (fully developed support). The score and justification for each item were obtained by arriving at a consensus among participating staff members. The mean was calculated from individual item scores to create a component score, and the mean of 6 component and integration scores formed the overall system score for the community health centre. The average time to complete the scale was 2 hours.

#### Quality of diabetes care

Quality of diabetes care was measured in terms of care processes and intermediate outcomes through auditing of medical records. The audit tool lists 28 services which the clinical guidelines in current use across the NT recommend for delivery at regular intervals for all people with diabetes (Table 1) [12]. A service was assessed as delivered if there was a record of delivery within the appropriate period preceding the audit. The overall adherence to delivery of scheduled services for each patient was calculated by dividing the sum of services delivered by 28 (the total number of scheduled services), and expressing this as a percentage.

**Table 1:** Adherence to delivery of scheduled services for study participants (N = 295)

**Process items**	**Scheduled interval (months)**	**% of patients receiving services**	95% CI*
**Basic measurement**			
Weight	3	47%	41%–53%
Height	Any time	32%	27%–38%
BMI	12	16%	12%–21%
Waist circumference	3	23%	18%–28%
BP	3	63%	57%–69%
**Eye check**			
Visual acuity	12	40%	35%–46%
Cataracts	12	28%	23%–34%
Fundi (dilated pupils)	12	34%	29%–40%
Ophthalmologist review	24	34%	29%–40%
**Feet check**			
Check done	3	20%	16%–25%
Sensation	3	9%	6%–13%
Peripheral pulses	3	8%	5%–12%
Pressure areas	3	7%	5%–11%
Infections	3	8%	6%–12%
**Laboratory investigations**			
BSL (finger prick or venous)	3	61%	55%–67%
HbA1c^†^	6	41%	35%–47%
Fasting lipids	12	27%	22%–33%
Total cholesterol	12	56%	50%–62%
Urine – Dipstix	3	20%	15%–25%
Creatinine	12	65%	59%–71%
ACR	12	54%	48%–59%
**Counselling/advice**			
Diet	3	15%	11%–19%
Activity	3	13%	9%–17%
Smoking	3	10%	7%–14%
Alcohol	3	9%	6%–13%
Diabetes medications	3	10%	7%–14%
**Immunisations**			
Flu vac.	12	54%	48%–59%
Pneumo vac.	5 yrs	73%	68%–78%

* 95% CIs were calculated adjusting for clustering by health centre.

† During the past 12 month period, 70% (95%CI: 64%–75%) of patients had HbA1c tested.

Intermediate outcomes of diabetes care include three measures: the values of the most recent HbA1c, blood pressure, and total cholesterol within 12 months prior to the audit. Control of these 3 measures is essential in preventing or delaying the onset of macro- and micro-vascular complications [13].

### Statistical analysis

Means and proportions were used to summarise normally distributed continuous and binomial data respectively, and 95% confidence intervals were calculated after adjustment for clustering by heath centre. As the ACIC result represented a score ranking from 0 to 11, nonparametric measures were used to describe ACIC results in terms of median, interquartile range, and range.

Multiple linear regression analysis was used to determine the independent association of each health system component with the overall adherence to delivery of scheduled services, with patient level variables (age and sex) treated as covariates.

The associations between system components and intermediate outcomes of diabetes care were assessed using multivariate probit regression [14]. This statistical procedure allows three dependent variables Y_1_(HbA1c control), Y_2 _(blood pressure control) and Y_3 _(total cholesterol control) to be jointly regressed on the same independent variables (6 system components and participants' age and sex) in one model. The dependent variables are dichotomous and defined as follows: Y_1 _= 1 if HbA1c level < 8.0%, and Y_1 _= 0 if HbA1c = 8.0% or no HbA1c tested within the past 12 months; Y_2 _= 1 if blood pressure < 140/90 mmHg, and Y_2 _= 0 if blood pressure ≥ 140/90 mmHg or no blood pressure checked within the past 12 months; Y_3 _= 1 if total cholesterol < 5.5 mmol/L, and Y_3 _= 0 if total cholesterol ≥ 5.5 mmol/L or no total cholesterol tested within the past 12 months. The associations with a single intermediate outcome, and the three as a group, were separately tested. All analyses were performed using Stata version 8.2 [15].

Ethics approval for the study was obtained from the Top End Health Research Ethics Committee, including the Indigenous sub-committee.

## Results

The records of 295 people with diabetes (116 males and 179 females) were included in the study. The average age of participants was 49 years (range 16–87). Diabetes duration averaged 6.5 years. Seventy-seven percent (95%CI: 71%–81%) of participants had attended their community health centres within the previous 3 months.

### ACIC scores for community health centre systems

**Figure 2:**
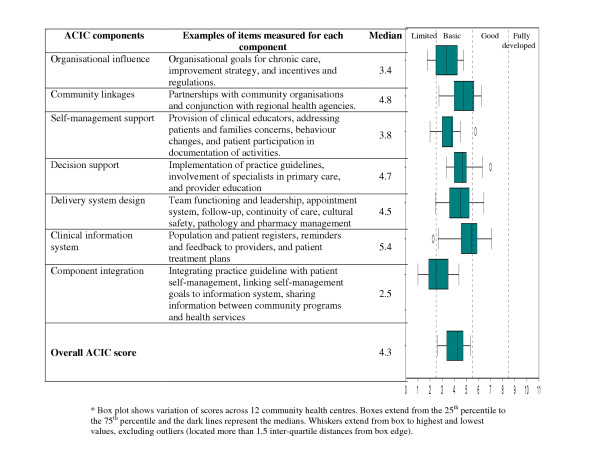
Assessment of Chronic Illness Care (ACIC) component scores for participating community health centres (N = 12).

The overall ACIC score ranged from 2.6 to 5.3 with a median of 4.3 (Figure 2). The median ACIC scores for system components ranged from 2.5 (component integration) to 5.4 (clinical information system). Community health centres had distinct areas of strengths and weakness as reflected in the qualitative justification in each system component and summarised below.

#### Organisational influence

five of twelve health centres included chronic disease care goals in their business plan and had one or more chronic disease coordinators in place. One health centre had an established continuous improvement program. Public health nurse positions, recently developed by the Department of Health and Community Services, were perceived by health service staff as an improvement strategy for chronic illness care. Six health centres reported use of Enhanced Primary Care Medicare claims as incentives for chronic care planning.

#### Community linkages

All participating health centres reported going out into the community to 'collect' people for specialist visits as a common activity. Some health centres worked together with other organisations and ran community-based programs such as 'school nutrition programs', 'healthy lifestyle promotional days' at the local store, 'healthy kids week', or 'tobacco prevention week'. However, all health service staff reported that acute care demands often prevented the development of community relationships that may have improved chronic care.

#### Self-management support

There was limited uptake and documentation of self-management activities in health centres, such as goal setting with clients and patient education. One-to-one education was the most commonly mentioned approach for the delivery of patient education. Peer or group education was seldom used.

#### Clinical decision support

Clinical guidelines (e.g. CARPA standard treatment manual) [12] were universally distributed to centres to facilitate clinical decision-making, and most health centres reported integration of these guidelines into routine care. Involvement of specialists in primary care was mainly through conventional referrals, and visiting specialist services to health centres were perceived generally as not frequent enough to meet needs.

#### Clinical delivery system

Most health centres suffered from a shortage of staff, and only six communities had a resident doctor. Few centres adopted planned visits for delivering multiple services to clients. All centres reported that systems for collecting and reporting of pathology specimens and for dispensing medication were in place. Most respondents perceived their centres to be delivering culturally safe and appropriate services.

#### Clinical information systems

Computerised information systems were installed in 11 health centres, with four different clinical information software systems. The remaining centre used a paper-based information system only. Recall systems were operational for eight health centres (6 computerised, 1 paper-based, and 1 with both). Most centres had organised and easily accessible patient records. Most information systems lacked the capacity (or were not used) to supply staff with population-based information on quality of chronic illness care.

#### Integration of system components 

The *integration of system components *was the least developed area. For example, the existing information system was not integrated with patient self-management in terms of documenting goals and activities; and there was limited sharing of information between out-of-clinic community programs and clinical services.

### Quality of diabetes care

Adherence to delivery of scheduled services varied across different categories of services (Table 1). Adherence was relatively higher for immunisation and laboratory investigations, followed by eye checks and basic measurement. Least attention was paid to feet checks and counselling services. The overall adherence to delivery of scheduled services for individuals averaged 31% (range 0–93%).

For intermediate outcomes (Table 2), the most recent value of HbA1c was below 8% for only about a quarter of participants, and most recent value of BP was less than 140/90 for about half of the participants.

**Table 2:** Intermediate outcomes of diabetes care for study participants (N = 295)

Intermediate outcomes*	**Mean ^†^(95%CI)**	**Proportion ^‡^(95% CI)**
HbA1c level (%)	9.3 (9.0–9.6)	
HbA1c < 8%		26% (21%–31%)
Systolic blood pressure (mmHg)	130 (127–133)	
Diastolic blood pressure (mmHg)	79 (78–81)	
Blood pressure <140/90 mmHg		54% (48%–59%)
Total cholesterol level (mmol/L)	4.9 (4.7–5.1)	
Total cholesterol <5.5 mmol/L		41% (35%–47%)

* The most recent readings in the past 12 months were used.

† Among patients receiving measurements.

‡ Patients not receiving measurements were treated conservatively as having outcomes beyond the cut points.

95% CI was calculated by adjusting for clustering by health centre.

### Associations between system ACIC scores and quality of diabetes care

Analysed by the linear regression modelling, each ACIC component was statistically associated with overall adherence to delivery of diabetes services (Table 3). After adjustment for other system components and individual variables, organisational influence, community linkages, and information system were identified as having independent associations with adherence to delivery of diabetes services. For example, an increase of 1 unit in the information system ACIC score was associated with an improvement of 4.5% in overall adherence to delivery of services.

**Table 3:** Association of health centre system components with overall adherence to delivery of scheduled services^§^

**Variables**	**Unadjusted Coefficients**	**95% CI**	Adjusted Coefficients*	**95% CI**
**Individual level variables**				
Age	-0.06	-0.23, 0.11	-0.11	-0.29, 0.07
Sex^†^	**5.10**	0.49, 9.70	5.63	-0.07, 11.33
**ACIC Components**				
Organisational influence	**6.27**	4.10, 8.44	**4.30**	0.92, 7.69
Community linkages	**4.60**	2.41, 6.79	**3.83**	1.89, 5.76
Self-management	**2.58**	0.25, 4.91	-2.30	-5.39, 0.79
Decision support	**2.75**	0.68, 4.83	-2.30	-5.09, 0.49
Delivery system	**2.30**	0.47, 4.14	-0.48	-3.96, 2.99
Information system	**3.87**	2.30, 5.44	**4.52**	0.70, 8.34
Integration^‡^	**2.94**	0.76, 5.11		

§ Estimated using multiple linear regression models.

* Adjusted for other variables in the table (except variable integration) and for clustering by health centre. The intercept for the linear regression is -7.25.

† Males are referent.

‡ Integration was excluded from adjusted analysis as it was correlated with other ACIC components and caused colinearity in multiple regression models.

Coefficients significant at 0.05 level are shown in bold.

The likelihood of each of HbA1c, blood pressure, and total cholesterol being below the specified cut point rose significantly with an increase in the organisational influence score (Table 4). Higher delivery system design and information system scores were associated with better blood pressure control and total cholesterol control respectively. Organisational influence, delivery system design and information system scores were also significantly associated with higher combined intermediate outcome scores.

**Table 4:** Association between scores for health centre system components and measures of intermediate outcomes of diabetes care

**Variables**	**HbA1c control (Y_1_)**	**Blood pressure control (Y_2_)**	**Total Cholesterol control (Y_3_)**	**Effect of independent variables on joint intermediate outcomes P value ^‡^**
		
	Adjusted Odds Ratios (95%CI) *			
**Individual level variables**				
Age	1.02 (0.99,1.03)	0.99 (0.98,1.01)	1.00 (0.99,1.02)	0.234
Sex^†^	1.24 (0.90,1.71)	1.27 (0.86,1.88)	1.19 (0.86,1.64)	0.068
**ACIC Components**				
Organisational influence	**1.47 (1.23,1.76)**	**1.66 (1.21,2.28)**	**1.22 (1.03,1.45)**	**0.000**
Community linkages	1.03 (0.91,1.17)	0.97 (0.76,1.22)	1.14 (0.96,1.35)	0.108
Self-management	0.97 (0.83,1.13)	0.85 (0.66,1.11)	0.89 (0.73,1.09)	0.053
Decision support	0.89 (0.78,1.02)	1.10 (0.88,1.38)	0.85 (0.72,1.01)	0.064
Delivery system	1.05 (0.89,1.23)	**1.42 (1.06,1.90)**	0.88 (0.74,1.06)	**0.000**
Information system	1.05 (0.89,1.23)	0.75 (0.54,1.03)	**1.27 (1.04,1.54)**	**0.004**

Y_1_, Y_2_, and Y_3 _are dichotomous variables (0,1), and having a value of 1 represents HbA1c < 8.0%, blood pressure < 140/90 mmHg, and total cholesterol < 5.5 mmol/L, respectively.

* Adjusted for other variables in the table and for clustering by health centre.

† Males are referent.

‡ Estimated using multivariate probit model. P values below 0.05/8 = 0.0063 (8 because there are 8 independent variables) are declared significant at 5% level.

Odds ratios significant at 0.05 level are shown in bold.

## Discussion

This study shows that participating Aboriginal community health centres in Australia had implemented basic systems to support chronic illness care, but there was considerable room for improvement in all system components. Stronger organisational influence and information system components were associated both with better performance in process of diabetes care and in intermediate outcomes. Additionally, community linkages were specifically related to better performance in process of care, and delivery system design was associated with better intermediate outcomes.

When compared with data from the USA [16], health centres had similar ACIC scores for community linkages, decision support, delivery system, and clinical information system, but lower scores for organisational influence and self-management support. In many respects the quality of diabetes care in participating health centres is also comparable with national and international data [17-19] For example, 70% of patients in our study had HbA1c tested in the past year and 26% had values less than 8% – almost identical to experience reported from the USA [19].

To our knowledge, this is the first study to demonstrate quantitative evidence regarding the importance of organisational influence (including goals for chronic care, improvement strategies, and incentives for care) on diabetes care. Wagner and colleagues reported their experience in the chronic condition Breakthrough Series, suggesting the removal of disincentives in practice encourages providers' in delivery of effective chronic illness care [7]. Financial incentives for diabetes care have been introduced to Australian general practice through Enhanced Primary Care (EPC) and Practice Incentive Program (PIP) [20,21]. However, only half of participating centres reported claiming for Medicare rebate using EPC items.

Our study shows better implementation of clinical information systems to be associated with both increased adherence to guideline-recommended processes of diabetes care and improved intermediate outcomes. An ideal information system has three important roles: 1) as a registry for a target population; 2) to provide reminders to primary care teams to comply with guidelines for care; and 3) to provide feedback measures relevant to quality of care [22]. Our data show the third role to be the least-developed area for current information systems in this study setting, and support the appropriateness of external clinical audit as a useful approach to address such system deficiencies [23,24]

The positive relation between delivery system design and intermediate outcomes of diabetes care is consistent with several previous studies [25,26] It is likely that health centres characterised by availability of resident doctors, active specialist outreach, and appropriate client follow-up offer more opportunity for intensive management that might contribute to better diabetes control. Given that many remote community health centres are staffed primarily by nurses and Aboriginal Health Workers (AHWs) and supported by visiting doctors [27], a feasible approach to improve delivery system design is to assign and strengthen nurses' and AHWs' roles in delivering routine care, and to ensure referral to medical practitioners for consultation and medication adjustment where appropriate [28]. Features of delivery system design may also be amenable to improving the relative under-utilisation of primary care services by Aboriginal men – a widely recognised phenomenon that is reflected in the study sample.

The apparent poor integration of system components in participating health centres also needs to be addressed in future system development, as isolated upgrading in one component without integrating with another may lead to an increase in costs but not in effectiveness. For example, computerised information systems can generate "pop-up" reminders for healthcare providers, but poor delivery system design characterised by unclear roles among health staff may result in no appropriate action being taken.

The cross-sectional study design limits the confidence with which the observed associations between health centre systems and processes and outcomes of diabetes care can be defined as causal. However, the findings suggest that the Chronic Care Model and the associated ACIC scale will be valuable in assessing and guiding the development of health centre systems in Aboriginal community settings. More research is needed to formally examine the reproducibility of the methods of assessing systems development, and to define the cause-effect relationship between healthcare systems and quality of diabetes care using longitudinal study designs. Such studies may also help clarify resource and management requirements for sustaining improvements in chronic illness care.

## Conclusion

The state of development of health centre systems is reflected in quality of care outcome measures for patients. The health centre systems assessment tool proves to be useful in describing the quality of clinical systems for the prevention and management of diabetes in Australian Aboriginal communities, and providing a guide for development of systems for improving diabetes care.

## Competing interests

The author(s) declare that they have no competing interests.

## Authors' contributions

DS participated in developing the study design, performed the statistical analyses, and drafted the manuscript. RB conceived and designed the study, supervised the data collection and analyses, provided a major role in revising the manuscript. CC participated in the study design and assisted in interpretation of findings. MD and AS developed questionnaires and performed the data collection. GR, JC, and TW participated in the development of study design, editing and revising the manuscript. All authors contributed to, have read and approved the final manuscript.

## Pre-publication history

The pre-publication history for this paper can be accessed here:



## Supplementary Material

Additional File 1 Assessment of Chronic Illness Care (ACIC scale) Click here for file

Additional File 2 Example: the score and justification for an item in the ACIC scale, which were made by health centre staff consensus Click here for file
