# Supplementary material for: Assessing health centre systems for guiding improvement in diabetes care
Source: BMC Health Serv Res. 2005 Aug 24;5:56. doi: 10.1186/1472-6963-5-56 (PMC1208882; doi:10.1186/1472-6963-5-56)
Supplement: Additional File 1 — Assessment of Chronic Illness Care (ACIC scale) [file 1472-6963-5-56-S1.doc]

# Additional file 1: **Assessment of Chronic Illness Care (ACIC scale)**

**1. Organisational influence**: Chronic illness management can be more effective if the organisation in which care is provided is oriented and led in a manner that allows for a focus on chronic illness care.

| **Limited *or no* support** | **Basic support** | **Good support** | **Fully developed support** |
| --- | --- | --- | --- |
| **1.1 Organisational commitment (goals) for chronic illness care………….. Score =** | | | |
| 0 1 2  …does not exist or there is little interest. | 3 4 5  …is reflected in vision statements and business plans, but no resources are specifically earmarked for chronic illness care. | 6 7 8  …is reflected in vision statement and business plans, and specific resources (dollars and personnel) have been allocated by senior leadership to implement it, but funding is still vulnerable and connected in some way to acute care, and discontinuity of staffing may still be an issue. | 9 10 11  …is part of the system’s long term planning strategy, long-term financing is assured, adequate equipment / educational resources / staff are secured, and specific people are held accountable for implementing, evaluating and updating the policy. |
| **Justification for score:** | | | |
| **1.2 Improvement Strategies for Chronic Illness Care………….. Score =** | | | |
| 0 1 2  …are not organised or supported, e.g. no systematic handling of errors and quality problems, no planning and review cycle established or supported by management. | 3 4 5  …utilises ad hoc approaches for targeted problems as they emerge; Some attempt at identifying and reporting quality problems but not necessarily followed through; quality improvement personnel may be employed but may not be fully active. | 6 7 8  …utilises improvement strategies for targeted problems; system for reporting and considering errors and quality problems is operational; some changes followed through; quality improvement personnel are employed who are actively involved in chronic illness care. | 9 10 11  …includes a proven improvement strategy and uses it proactively in meeting organisational goals; errors and care problems are reported, studied, and appropriate changes put into action systematically, with regular review; quality improvement personnel use designated resource to integrate quality improvement programs into routine care. |
| **Justification for score:** | | | |
| **1.3 Incentives (and Regulations) for Chronic Illness Care ………….. Score =** | | | |
| 0 1 2  Minimal awareness or availability of incentive funds to support chronic illness care, e.g. Enhanced Primary Care item claims are not made. | 3 4 5  Incentive funds are available, but are applied for and used in ad hoc approach, e.g. Enhanced Primary Care claims are submitted and used to influence utilisation of chronic care. | 6 7 8  Incentive funds are applied for and used regularly, e.g. regular reimbursements from Enhance Primary Care claims are available, and used to support patient care goals. | 9 10 11  Incentive funds are regularly received to motivate and empower providers to support ongoing health care programs and to achieve maximum effect. |
| **Justification for score:** | | | |

## Organisational influence score (sum of item scores / 3): ________

**2. Community linkages:** linkages between the health care organisation and community resources (and external service providers) play important roles in the management of chronic illness.

| **Limited *or no* support** | **Basic support** | **Good support** | **Fully developed support** |
| --- | --- | --- | --- |
| **2.1 Linking patients to outside resources………….. Score =** | | | |
| 0 1 2  …is not done systematically. | 3 4 5  …is limited to a list of identified community or outside resources and some may be used but not to maximum effect. | 6 7 8  …is accomplished through a person or resource responsible (or service agreement) ensuring providers and patients make good use of outside resources/ service providers. | 9 10 11  …is accomplished through active coordination between the health system, community service agencies and patients. |
| **Justification of score:** | | | |
| **2.2 Partnerships and activities with Community based organisations………….. Score =** | | | |
| 0 1 2  …do not exist. | 3 4 5  …are being considered but have not yet been implemented. | 6 7 8  …are formed to develop supportive programs, (projects and policies) with the health care clinic. | 9 10 11  …are actively sought to develop formal supportive programs across the entire system. |
| **Justification of score:** | | | |
| **2.3 Working out in the community………….. Score =** | | | |
| 0 1 2  Clinic staff time spent all of their time within the Health Centre. | 3 4 5  Clinic staff go out into the community to collect people, and do some work out in the community. | 6 7 8  Designated person to work out in the community with organisations, families, and individuals in health promotion/health education/& emotional support; community involvement but low levels of control; | 9 10 11  Work in community is reported on; work plan is in place; Health Centre and organisation provide resources and male and female designated staff to work outside the Health Centre in a creative manner; high levels of community involvement in planning and control; working with groups and families. |
| **Justification of score:** | | | |
| **2.4 Coordination with other agencies………….. Score =** | | | |
| 0 1 2  Does not coordinate chronic illness guidelines, measures or care resources with regional agencies. | 3 4 5  Would consider some degree of coordination of guidelines, measures of care resources but have not yet implemented changes. | 6 7 8  Currently coordinates guidelines, measures or care resources with regional health agencies in one or two chronic illness areas. | 9 10 11  Currently coordinates chronic illness guidelines, measures and resources in conjunction with regional health agencies for most chronic illnesses. |
| **Justification of score:** | | | |

## Community Linkages Score (sum of item scores / 4) _________

**3. Self-Management Support:** effective self-management support can help patients and families cope with the challenges of living with and treating chronic illness and reduce complications and symptoms.

| **Limited *or no* support** | **Basic support** | **Good support** | **Fully developed support** |
| --- | --- | --- | --- |
| **3.1 Assessment and documentation of self-management needs and activities ………….. Score =** | | | |
| 0 1 2  …are not done. | 3 4 5  …are expected, may be done but with minimal client involvement; one way process. | 6 7 8  …are completed collaboratively by patients and providers in a standardised manner. | 9 10 11  …are regularly completed and assessments are recorded in standardised form linked to a treatment plan available to practice and patients. |
| **Justification of score:** | | | |
| **3.2 Self-management education and support………….. Score =** | | | |
| 0 1 2  …is limited to the distribution of information (pamphlets, booklets). | 3 4 5  …is available by referral to self-management classes (or groups) or by distributing tools (eg guidelines, weight logs) to clients. | 6 7 8  …is provided on an as-needed (referral) basis by trained staff, along with tools to help clients adhere to the programs and track progress. | 9 10 11  …is provided by staff trained in client empowerment and problem-solving and behavioural support methodologies, along with culturally appropriate tools to help clients understand their illness, adhere to programs, and track progress. |
| **Justification of score:** | | | |
| **3.3 Addressing concerns of patients and families………….. Score =** | | | |
| 0 1 2  …is not consistently done. | 3 4 5  …is provided for specific patients and families through referral. | 6 7 8  …is encouraged, and peer support, groups, and mentoring programs are available. | 9 10 11  …is an integral part of care and includes systematic assessment and routine involvement in peer support, groups or mentoring programs. |
| **Justification of score:** | | | |
| **3.4 Effective behaviour change interventions……….. Score =** | | | |
| 0 1 2  …are not available. | 3 4 5  …are limited to the distribution of pamphlets, booklets or other written information. | 6 7 8  …are available only by referral to specialised centres staffed by trained personnel. | 9 10 11  … are readily available within the health centres as an integral part of routine care. |
| **Justification of score:** | | | |

# **Self-Management Support score (sum of item scores / 4) _______**

## 4. Decision Support: effective chronic illness management assures that providers have access to evidence-based information necessary to care for patients--decision support. This includes evidence-based practice guidelines or protocols, speciality consultation, and provider education.

| **Limited or no support** | **Basic support** | **Good support** | **Fully developed support** |
| --- | --- | --- | --- |
| **4.1 Evidence Based Guidelines………….. Score =** | | | |
| 0 1 2  …are not available. | 3 4 5  …are available but are not integrated into care delivery. | 6 7 8  …are distributed, integrated into care delivery, and supported by provider education. | 9 10 11  …are available, regularly updated by the organisation, supported by provider education and integrated into care through reminders, and staff activated to systematically apply standards. |
| **Justification of score:** | | | |
| **4.2 Involvement of specialists in improving primary care………….. Score =** | | | |
| 0 1 2  …is primarily through traditional referral. | 3 4 5  …is achieved through specialist leadership to enhance the capacity of the overall system to routinely implement guidelines. | 6 7 8  …includes specialist leadership and designated specialists who provide primary care team training. | 9 10 11  …includes ongoing specialist leadership and collaboration in improving the care of primary care patients. |
| **Justification of score:** | | | |
| **4.3 Provider education for chronic illness care ………….. Score =** | | | |
| 0 1 2  … is provided sporadically. | 3 4 5  … is provided systematically through traditional CME methods. | 6 7 8  … is provided systematically using more innovative and tailored methods (e.g. academic detailing). | 9 10 11  … is provided systematically using more innovative methods for the entire practice teams and includes population-based management, and self-management support. |
| **Justification of score:** | | | |

# **Decision Support score (sum of item scores / 3) _______**

**5. Delivery System Design:** evidence suggests that effective chronic illness management involves more than simply adding additional interventions to a current system focused on acute care. It may necessitate changes to the organisation of practices that impact on provision of care.

| **Limited *or no* support** | **Basic support** | **Good support** | **Fully developed support** |
| --- | --- | --- | --- |
| **5.1 Practice team functioning: ………….. Score =** | | | |
| 0 1 2  …is not addressed; Irregular or no team meetings; Obvious lack of cohesion, coordination and poor communication. | 3 4 5  …is addressed by assuring the availability of individuals with appropriate training in key elements of chronic illness care. | 6 7 8  …is assured by regular team meetings to address guidelines, roles and accountability, and problems in chronic illness care. | 9 10 11  …is assured by teams who meet regularly and have clearly defined roles including patient self-management education, proactive follow-up, and resource coordination and other skills in chronic illness care. |
| **Justification of score:** | | | |
| **5.2 Leadership in chronic disease care:………….. Score =** | | | |
| 0 1 2   - …is not recognised locally or by the system. | 3 4 5  …is assumed by the organisation to reside in existing organisational roles. | 6 7 8  …is assured by the appointment of a team leader but the role in chronic disease is not ‘protected’ | 9 10 11  …is guaranteed by the appointment of a team leader or disease coordinator whose role is assured and in turn assures that roles and responsibilities for chronic illness care are clearly defined and protected. |
| **Justification of score:** | | | |
| **5.3 Appointment System: ………….. Score =** | | | |
| 0 1 2  …can be used to schedule acute care visits, follow-up and preventive visits. | 3 4 5  …assures scheduled follow-up with chronically ill patients. | 6 7 8  …is flexible and can accommodate innovations such as customised visit length or group visits. | 9 10 11  …includes organisation of care that facilitates the patient seeing multiple providers in a single visit (e.g. Chronic disease days or weeks with presence of specialist providers). |
| **Justification of score:** | | | |
| **5.4 Follow-up: ………….. Score =** | | | |
| 0 1 2  …is scheduled by patients or providers in an ad hoc fashion. | 3 4 5  …is scheduled by the practice in accordance with Guidelines. | 6 7 8  …is assured by the practice team by monitoring patient utilisation and by a quality recall system (Autonomy of client transport is also considered). | 9 10 11  …is customised to patient needs, varies in intensity and methodology (phone, in person) and assures guideline follow-up. |
| **Justification of score:** | | | |
| **5.5 Planned Visits for Chronic Illness Care: ………….. Score =** | | | |
| 0 1 2  …are not used. | 3 4 5  …are occasionally used for complicated patients. | 6 7 8  …are an option for interested patients. | 9 10 11  …are used for all patients and include regular assessment, preventive interventions and attention to self-management support. |
| **Justification of score:** | | | |
| **5.6 Continuity of Care: ………….. Score =** | | | |
| 0 1 2  …is not a priority. | 3 4 5  …has some priority, depends on written and phone communication between primary care providers and specialists, allied health, hospital etc. | 6 7 8  …between primary care providers and specialists and other relevant providers is a priority but not implemented systematically. | 9 10 11  …is a high priority and all chronic disease interventions including active coordination between primary care, specialists and other relevant groups. |
| **Justification of score:** | | | |
| **5.7 Client Access /Cultural competence issues: ………….. Score =** | | | |
| 0 1 2  …is not considered: clinic not well sited; gender issues not addressed; competency principles are not in place; assumption that all people are alike and that what works in one culture should work in another . | 3 4 5  …have been considered to an extent: some action directed at making the Health Centre appropriate for indigenous client comfort and use; some thought given to cultural competence which encourages learning and understanding of new ideas and solutions to improve performance or services. | 6 7 8  A range of factors considered: translators available (when necessary); confidentiality in place; some attempt at structural appropriateness; gender considerations obviously implemented; cultural competence achieved through actively seeking advice and consultation and commitment to incorporating new knowledge and experiences into a wider range of practice. | 9 10 11  Highly appropriate level of cultural access and competence achieved: health centre designed and built and managed for maximum accessibility to all groups; confidentiality in place; cultural safety assured; proficient cultural competence involving holding cultural differences and diversity in high esteem, pro-activity regarding cultural differences, and promotion of improved cultural relations. |
| **Justification of score:** | | | |
| **5.8 Well functioning pathology system: ………….. Score =** | | | |
| 0 1 2  …is not assured, range of problem issues make it dysfunctional. | 3 4 5  …routine tests and opportunistic checks are done sporadically, transport, labelling, filing, follow-up in place but may be disorganised or below standard; may be issues re lack of confidentiality. | 6 7 8  Screening and follow-up standards generally good; system of taking, transporting, receiving path results works well; confidentiality considered; filing and follow-up generally adequate. | 9 10 11  System working to a high standard; confidentiality assured; results double-checked, shared and filed for accessibility; necessary follow-up assured; clients informed of results, recalls automatic. |
| **Justification of score:** | | | |
| **5.9 Well functioning Pharmacy system: ………….. Score =** | | | |
| 0 1 2  …not assured, does not exist, or much work required. | 3 4 5  Systems to encourage compliance only partly supported; storage area exists; stock-control system present; no pharmacy in-services; no guidelines for dispensing; labels hand-written; expiry dates may be problem; waste a problem. | 6 7 8  Ordering working well; storage area meets standards for dispensing; waste minimised; expiry dates controlled; occasional in-services and audits; drugs labelled to standard; pharmaceutical guidelines only partly applied. | 9 10 11  Regular in-services (for staff) and stock-takes; blister packs used; computer scripts; storage and dispensing area to standard; no wasted drugs; medications checked for adjustment regularly; dispensary has copy of recall list; pharmaceutical guidelines adhered to; drug compatibility checks assured. |
| **Justification of score:** | | | |

**Delivery System Design score (sum of item scores / 9) _______**

**6. Clinical Information Systems:** timely, useful information about individual patients and populations of patients with chronic conditions is a critical feature of effective management, especially that employing population-based approaches.

| **Limited *or no* support** | **Basic Support** | **Good Support** | **Fully developed support** |
| --- | --- | --- | --- |
| **6.1 Register of community members (Health Centre population list): ………….. Score =** | | | |
| 0 1 2  No population list. | 3 4 5  Population list present but is out of date, or disorganised. | 6 7 8  Population list reviewed every now and then; some idea of who is coming and going from the community. | 9 10 11  Population list up to date and reviewed regularly; policy for deciding on visitors and residents in place; high degree of knowledge about who is in the community and who has left. |
| **Justification of score:** | | | |
| **6.2 Register of patients with specific conditions: ………….. Score =** | | | |
| 0 1 2  …is not available. | 3 4 5  …includes name, diagnosis, contact information and date of last contact either on paper or in a computer database. Information about service delivery to subgroups of patients can only be obtained with special efforts or additional programs. | 6 7 8  …allows queries to sort sub-populations by clinical priorities. Information about service delivery to subgroups of patients can be obtained upon request but is not routinely available. | 9 10 11  …is tied to guidelines which provide prompts and reminders about needed services. Information about service delivery to subgroups of patients is provided routinely to providers to help them deliver planned care. |
| **Justification of score:** | | | |
| **6.3 Reminders to providers: ………….. Score =** | | | |
| 0 1 2  …are not available. | 3 4 5  …include general notification of the existence of a chronic illness, but do not describe needed services at time of encounter. | 6 7 8  …include indications of needed service for populations of patients through periodic reporting. | 9 10 11  …include specific information for the team about guideline adherence at the time of individual patient encounters. |
| **Justification of score:** | | | |
| **6.4 Feedback: ………….. Score =** | | | |
| 0 1 2  …is not available or is non-specific to the team. | 3 4 5  …is provided at infrequent intervals and not fed back directly or is fed back inappropriately or inadequately. | 6 7 8  …occurs at frequent enough intervals to monitor performance and is specific to the team’s population. | 9 10 11  …is timely, specific to the team, routine and personally delivered by a respected opinion leader to improve team performance. |
| **Justification of score:** | | | |
| **6.5 Patient Treatment Plans: ………….. Score =** | | | |
| 0 1 2  …are not expected, and not in use. | 3 4 5  …are achieved through a standardised approach, but do not appear in all files, and are not designed or completed to sufficient standard. | 6 7 8  …are established collaboratively for all clients and include self management as well as clinical and client goals but may not have been signed by client. | 9 10 11  …are established collaboratively (with client) and include self management as well as clinical management. Follow-up occurs at guideline intervals and directs care at every point of service. |
| **Justification of score:** | | | |
| **6.6 Records and filing system: ………….. Score =** | | | |
| 0 1 2  Files disorganised; hard to find; poorly kept; no summary sheets; co-morbidities not in front of file. | 3 4 5  Files organised, but inefficient system; some mixing of residents and non-residents; files generally legible; files may not be promptly re-filed after use; some files may have summary sheets at front; some co-morbidities in front of file. | 6 7 8  Files well organised and accessible; cabinets kept locked; clients have some access to their files; files legible; files mostly returned quickly after use; summary sheets and Care Plans in most files; co-morbidities generally in front of file. | 9 10 11  Files well organised and rapidly accessible (numerical and colour coding); cabinets kept locked; clients have access to their files; files always returned quickly after use; all staff trained in system; filing procedure present; summary sheets and care plans completed by all staff to high standard in all files; co-morbidities in front of all files. |
| **Justification of score:** | | | |

**Clinical Information System score (sum of item scores / 6) ________**

**7. Integration of Chronic Care Model Components:** effective systems of care integrate and combine all elements of the Chronic Care Model; e.g. linking patients’ self-management goals to information systems/registries.

| **Limited *or no* support** | **Basic Support** | **Good Support** | **Fully developed support** |
| --- | --- | --- | --- |
| **7.1 Integration of information systems with self-management support: ………….. Score =** | | | |
| 0 1 2  Information systems do not include patient self-management goals. | 3 4 5  Information systems include results of patient assessments (e.g., functional status rating; readiness to engage in self-management activities), but no goals. | 6 7 8  Information systems include results of patient assessments, as well as self-management goals that are developed using input from the practice team/provider and patient. | 9 10 11  Information systems include results of patient assessments, as well as self-management goals that are developed using input from the practice team and patient; and prompt reminders to the patient and/or provider about follow-up and periodic re-evaluation of goals. |
| **Justification of score:** | | | |
| **7.2 Integration of community programs with health centres: ………….. Score =** | | | |
| 0 1 2  Community program do not provide feedback to the health centres about patients’ progress in their programs. | 3 4 5  Community programs provide feedback at joint meetings between the community and health centres about patients’ progress in their programs. | 6 7 8  Community programs provide regular feedback to the health centre using formal mechanisms about patients’ progress. | 9 10 11  Community programs provide regular feedback to the health centre about patients’ progress, and incorporate input from the health staff into programs to better meet the needs of patients. |
| **Justification of score:** | | | |
| **7.3 Integration Organisational Planning with other system components: ………….. Score =** | | | |
| 0 1 2  Organisational planning does not involve a population-based approach. | 3 4 5  Organisational planning uses data from information systems to plan care. | 6 7 8  Organisational planning uses data from information systems to proactively plan population-based care, including the development of self-management programs and partnerships with community resources. | 9 10 11  Organisational planning uses systematic data and input from practice teams to proactively plan population-based care, including the development of self-management programs and community partnerships, which include a built-in evaluation plan to determine success over time. |
| **Justification of score:** | | | |
| **7.4 Integration of information systems with delivery system design: ………….. Score =** | | | |
| 0 1 2  …is not ensured. | 3 4 5  …is sporadically done, usually for appointments only. | 6 7 8  …is ensured by assigning responsibilities to specific staff (e.g., nurse case manager) and by having a system in place to allow and support the task fully. | 9 10 11  …is ensured by presence of a well designed and functional system and assignment of responsibilities to specific staff (eg nurse case manager) who use the registry and other prompts to coordinate with patients and the entire practice team. |
| **Justification of score:** | | | |
| **7.5 Integration of decision support with self-management support: ………….. Score =** | | | |
| 0 1 2  Guidelines for chronic illness care are not shared with patients. | 3 4 5  Guidelines are given to patients who express a specific interest in self-management of their condition. | 6 7 8  Guidelines are provided for all patients to help them develop effective self-management or behaviour modification programs, and identify when they should see a provider. | 9 10 11  Guidelines are reviewed by the practice team with the patient to devise a self-management or behaviour modification program consistent with the guidelines that takes into account patient’s goals and readiness to change. |
| **Justification of score:** | | | |

# **Integration score (sum of item scores / 5) __________**

## Scoring Summary

## (Bring forward the scores at the end of each section to this page)

| System components | Scores |
| --- | --- |
| Organisational Influence |  |
| Community Linkages |  |
| Self-Management Support |  |
| Decision Support |  |
| 1. Delivery System Design |  |
| Clinical Information Systems |  |
| Integration |  |

**Overall system score (sum of component scores / 7) ________**
