# Supplementary material for: Assessing health centre systems for guiding improvement in diabetes care
Source: BMC Health Serv Res. 2005 Aug 24;5:56. doi: 10.1186/1472-6963-5-56 (PMC1208882; doi:10.1186/1472-6963-5-56)
Supplement: Additional File 2 — Example: the score and justification for an item in the ACIC scale, which were made by health centre staff consensus [file 1472-6963-5-56-S2.doc]

**Additional file 2**

**Example: the score and justification for an item in the ACIC scale, which were made by health centre staff consensus**

## 4. Decision Support: effective chronic illness management assures that providers have access to evidence-based information necessary to care for patients--decision support. This includes evidence-based practice guidelines or protocols, speciality consultation, and provider education.

| Limited or no support | Basic support | Good support | Fully developed support |
| --- | --- | --- | --- |
| 4.1 Evidence Based Guidelines………….. **Score = 9** | | | |
| 0 1 2  …are not available. | 3 4 5  …are available but are not integrated into care delivery. | 6 7 8  …are distributed, integrated into care delivery, and supported by provider education. | 9 10 11  …are available, regularly updated by the organisation, supported by provider education and integrated into care through reminders, and staff activated to systematically apply standards. |
| **Justification of score:**   - Each staff is given a copy of CARPA manual when employed, which is a collection of clinical guidelines developed by Central Australian Rural Practitioners Association (CARPA). - Regular in-services education and induction of new staff are centred on CARPA manual. - CARPA manual (fourth updated edition) workshop was attended by staff. - Procedures in policy folder reinforce the use of CARPA. - Computer system supports the use of guidelines but is not accessed by all staff. | | | |
